# Perceptions of the Role of West Virginia’s Cooperative Extension Service in Tobacco Control Coalitions

**DOI:** 10.3389/fpubh.2016.00083

**Published:** 2016-05-06

**Authors:** Donald Reed, Dana Lester, Kathy Danberry, Peggy Lambert Fink, Sherry Owens

**Affiliations:** ^1^West Virginia University, Morgantown, WV, USA; ^2^West Virginia Division of Tobacco Prevention, Charleston, WV, USA; ^3^West Virginia University Institute of Technology, Montgomery, WV, USA

**Keywords:** tobacco, coalitions, West Virginia

## Abstract

Despite the fact that the consequences of tobacco use are well identified and known, it remains the single most preventable cause of disease and death in the United States. In West Virginia alone, the adult prevalence of cigarette smoking is 26.8%. This study researches the perceptions of the Cooperative Extension Service’s involvement and role in county-level coalitions that address tobacco use in West Virginia. The research findings provide practical areas to increase the role of the Extension Service in these vital efforts to save lives, reduce economic hardships on families, and reduce the health-care burden on the state government.

## Introduction

Despite the fact that the consequences of illness, disease, and death from tobacco use are well identified and known, it remains the single most preventable cause of disease and death in the United States (1). In West Virginia, the adult prevalence of cigarette smoking, currently at 26.8%, is the highest of any of the 50 states (2). Smokeless tobacco use also remains high among high school males at 24.8% and adult males at 15.5% (2). Comparable with other health disparities, tobacco-related disparities are, to some extent, caused and perpetuated by social determinates of health, such as poverty, environmental threats, inadequate access to healthcare, inadequate healthcare, individual behavior choices, cultural norms, and education inequalities.

To address the burden of tobacco use, the West Virginian Division of Tobacco Prevention funds a regional Tobacco Prevention Coalition Network that assists the local tobacco control coalition in each county. These coalitions comprise local tobacco control advocates, community leaders, and health-care experts, and each coalition determines its own focus and is guided by the Centers for Disease Control’s (CDC) Best Practices in Tobacco Control recommendations.

Current research suggests that community coalitions are vital to tackle health disparities by addressing social issues of health using multiple interventions at multiple levels (3). Tobacco control coalitions work to accomplish the founding mission mandates of the Cooperative Extension Service: community leadership and improvement in quality of life. Of major importance is that the community coalitions work across all sectors of a community.

The coalition should resemble a sliced onion in practice: Bronfenbrenner’s model of ecological model of development conceives youth and adult development as a function of interactions between individuals and the contexts in which they live. According to the *Journal of Extension*, “longevity and size of the group influence a coalition’s success at reaching more people in the community and the importance of having coalitions made up of varied members is vital to success” (4).

## Cooperative Extension Service

The Cooperative Extension Service is a nationwide, non-credit educational network. Each U.S. state and territory has a state office at its land-grant university and a network of local or regional offices that house university faculty to focus on youth development, agriculture, families, and health.

This service was founded to help all Americans achieve healthier lifestyles. The key factor to this mission is the translation of evidence-based programing to the citizens of all ages in each state. The service’s 4-H Youth Development program has a healthy living mandate that addresses tobacco prevention by increasing young people’s mindfulness, skills, and experiences to make lifelong healthy choices (5). Indeed, 4-H Youth Development program provides an opportunity for county-level coalitions to reach young people through already established programs without reinventing the wheel, thereby allowing for shared resources in these times of tight budgets (6, 7).

The Cooperative Extension Service aims to create meaningful learning experiences for young people to prevent or intervene in these high-risk behavior areas, across a variety of contexts. For example, the Healthy Living Strategic Framework clearly identifies expected outcomes related to the main areas of health-risk behaviors (5). One way to make evidence-based programing more accessible to young people in accordance with the Healthy Living Mission is to leverage the resources with partners and experts in the communities, the land-grant institutions, and the state health departments, which also addresses the key-risk behavior areas, such as tobacco use. For example, the Ohio State University Cooperative Extension Service has been focusing on tobacco control within schools and communities for almost 25 years (8).

Since the Cooperative Extension Service is funded through West Virginia University with an Extension Service Agent in every county and since the work of tobacco control coalitions mirrors the 4-H Youth Development Healthy Living Mission Mandate, you would assume that the service is a member of every county-level tobacco control coalition in West Virginia. However, this is far from the case. From the researcher’s initial observations and discussions with tobacco control professionals and volunteers, the Cooperative Extension Service is a missing partner in most county-level tobacco control coalitions in West Virginia.

## Purpose of Research

This study investigates current perceptions of the Extension Service’s role in health promotion at the local/county level. The specific aims of this study are to measure West Virginian coalitions’ perceptions of the Extension Service’s involvement in their local coalitions and to make recommendations for the Extension Service’s role and usefulness in local coalitions.

## Methods

The commercial service SurveyMonkey was used to create the survey, which was pilot tested with three county coalitions and revised based on the feedbacks and suggestions from a small group of Extension Service faculty county coalition members, the WVU Prevention Research Center, and the Break Free Alliance. The WVU Institutional Review Board designated this research as exempt from review. Participants consented by voluntarily participating in the study.

Survey questions were developed to establish the scope and focus of the county-level coalitions; the scope of focus of involvement of Cooperative Extension Service in the county-level coalitions; and to establish common themes of types of assistance the coalitions’ need to be successful in their work.

A survey consisting of 14 multiple-choice and open-ended questions was designed to assess the following information: the geographic coverage area of the coalition, description of the coalition’s focus area, participants’ role in the coalition, coalition’s focus areas, types of tobacco controls that coalitions address, involvement and role of the Extension Service in the coalition, priority populations on which the coalition concentrates, community’s smoking/tobacco policy, and units of the Extension Service involved in the local coalition. It also examined the tobacco issues that the Extension Service faculty and staff work to address within the coalition and the areas in which the coalition needs assistance in addressing.

Community coalitions in West Virginia that address tobacco use were identified through their involvement with the West Virginian Division of Tobacco Prevention or the West Virginian Community Anti-Drug Association. The paid staff or chairs of the coalitions identified were asked to complete the survey voluntarily. The survey was distributed to the coalition’s staff or chair *via* email. Respondents were from 38 coalitions, covering 69% of West Virginia’s county coalitions that address tobacco use.

## Results

Community Anti-Drug Coalitions of America is a principal advocacy and training service for organizations that address tobacco use, although only 22% of coalitions (*n* = 8) are members, since most are county-level coalitions (94%; *n* = 34). The two primary focus areas include “Mixed” (both urban and rural areas) (47%; *n* = 17) and “Rural” (30%; *n* = 11).

A total of 48% (*n* = 13) of the coalitions addressed tobacco use as part of a broader agenda or goal and 44% (*n* = 12) addressed tobacco use as the sole focus of their work. The top three tobacco control efforts focus on youth tobacco prevention, adult tobacco cessation, and tobacco-free or smoke-free outdoor policies.

When asked if an Extension Service faculty member or staff member was part of the coalition, only 22% (*n* = 8) answered “Yes.” Of those coalitions that answered “Yes” to Extension Service involvement in the coalition, 57% identified the involvement of the 4-H Youth Development Unit (*n* = 4), 28% identified the involvement of the Families and Health Unit (*n* = 3), and 14% identified the involvement of the Agriculture and Natural Resources Unit (*n* = 1). A total of 42% identified Extension Service faculty and staff as part of the Coalition Leadership.

The coalitions were then asked “on which areas does your coalition need assistance to address tobacco use as part of your strategic objectives,” and the top three answers were, how to be an effective advocate for policy change: 74% (*n* = 26), involving and influencing parents: 60% (*n* = 21), and organizing your community coalition/taskforce: 57% (*n* = 20). Other identified areas of needed assistance included working with the media, grant writing, and helping coalition members understand the essentials of youth development.

The research was limited by the small number of county coalitions that responded to the study and the sole focus on West Virginia. Nevertheless, the research provides a starting point to look at how other Cooperative Extension Service’s in other states interact with tobacco control partners and coalitions.

## Discussion

Tobacco control coalitions are one of the most cost-effective and efficient strategies for achieving change at the local level through advocacy, education, community mobilization, policy development, and program implementation (9). According to the CDC’s Best Practices in Tobacco Control, in order for healthy communities to be achieved, we must empower local community coalitions to address tobacco use from a grassroots level (including smoking bans and increasing tobacco taxes), implement a state-level mass media campaign that targets the root causes of tobacco use in a culturally appropriate manner, and support cessation services by backing state tobacco quit lines, implementing health systems change around tobacco, and expanding insurance coverage to include tobacco cessation (10).

Tobacco control efforts fit directly into the founding mission of the Extension Service and into the 4-H Mission Mandate of Healthy Living. Tobacco control efforts give the Extension Service the ability to change the health of the individual, to improve the economic status of the family, and to reduce the burden of preventable health-care costs upon society. The Extension Service should focus on a smokeless tobacco prevention curriculum aimed at elementary students, which would fill a gap in West Virginian tobacco control programing. This focus would also address the coalition’s identified needs of assistance in “involving and influencing parents” and “helping coalition members understand the essentials of youth development.”

The Extension Service faculty and staff in West Virginia need to be more involved in this vital issue, which is being addressed in every county at the county level. The study shows that when Extension Service faculty and staff are involved in these coalitions, they serve in leadership roles. Those faculty and staff already involved in local coalitions could serve as catalysts and mentors to get other faculty and staff involved. The areas identified as in need of assistance would be a great starting point for the Extension Service faculty and staff to become involved in their county-level coalitions.

In a larger context, this shows an area of collaboration that could exist between the West Virginia Division of Tobacco Prevention, the West Virginia University Extension Service, and the West Virginia University School of Public Health. Upon achieving this collaboration, it could serve as a model for other land-grant institutions when tackling tobacco control issues within their state. The next steps in our efforts are to survey coalitions nationwide to see if Cooperative Extension Service is helping county-level coalitions across the nation.

## Author Contributions

DR: literature review, survey design, implementation, and article writing; DL: analysis and article writing; PF: adviser and article writing; KD: adviser and review; and SO: analysis and article writing.

## Conflict of Interest Statement

The authors declare that the research was conducted in the absence of any commercial or financial relationships that could be construed as a potential conflict of interest.
